# Biodegradable Nanoparticles-Loaded PLGA Microcapsule for the Enhanced Encapsulation Efficiency and Controlled Release of Hydrophilic Drug

**DOI:** 10.3390/ijms22062792

**Published:** 2021-03-10

**Authors:** Suji Ryu, Seungyeop Park, Ha Yeon Lee, Hyungjun Lee, Cheong-Weon Cho, Jong-Suep Baek

**Affiliations:** 1 Department of Bio-Health Convergence, Kangwon National University, Chuncheon 24341, Korea; 202016297@kangwon.ac.kr; 2 Department of Herbal Medicine Resource, Kangwon National University, 346 Hwangjo-gil, Dogye-eup, Samcheok-si 25949, Gangwon-do, Korea; sygo1113@naver.com (S.P.); dlgkdus33@naver.com (H.Y.L.); S92sky@naver.com (H.L.); 3 Institute of Drug Research and Development, College of Pharmacy, Chungnam National University, 99 Daehak-ro, Yuseong-gu, Daejeon 34134, Chungcheong, Korea

**Keywords:** alginate, chitosan, PLGA, hydrophilic drug encapsulation, microcapsule, controlled release, nanoencapsulation

## Abstract

Recently, nano- and micro-particulate systems have been widely utilized to deliver pharmaceutical compounds to achieve enhanced therapeutic effects and reduced side effects. Poly (DL-lactide-co-glycolide) (PLGA), as one of the biodegradable polyesters, has been widely used to fabricate particulate systems because of advantages including controlled and sustained release, biodegradability, and biocompatibility. However, PLGA is known for low encapsulation efficiency (%) and insufficient controlled release of water-soluble drugs. It would result in fluctuation in the plasma levels and unexpected side effects of drugs. Therefore, the purpose of this work was to develop microcapsules loaded with alginate-coated chitosan that can increase the encapsulation efficiency of the hydrophilic drug while exhibiting a controlled and sustained release profile with reduced initial burst release. The encapsulation of nanoparticles in PLGA microcapsules was done by the emulsion solvent evaporation method. The encapsulation of nanoparticles in PLGA microcapsules was confirmed by scanning electron microscopy and confocal microscopy. The release profile of hydrophilic drugs can further be altered by the chitosan coating. The chitosan coating onto alginate exhibited a less initial burst release and sustained release of the hydrophilic drug. In addition, the encapsulation of alginate nanoparticles and alginate nanoparticles coated with chitosan in PLGA microcapsules was shown to enhance the encapsulation efficiency of a hydrophilic drug. Based on the results, this delivery system could be a promising platform for the high encapsulation efficiency and sustained release with reduced initial burst release of the hydrophilic drug.

## 1. Introduction

Hypertension occurs when there is an increase in the pressure within the artery. It is a persistent medical condition where the blood pressure is elevated at or above 140/90 mmHg [1]. There are no initial symptoms, but sustained hypertension will increase the risk for coronary heart disease, stroke, and chronic kidney disease [2]. There are two classifications of hypertension, which are primary hypertension and secondary hypertension. Primary hypertension covers up to 90–95% of the cases where there is no obvious underlying cause. The remaining 5–10% of cases belong to secondary hypertension where there is an identifiable cause [1]. Lifestyle changes, environmental factors, age, and dietary and genetic susceptibility are some factors contributing to hypertension. Hypertension can be treated or ameliorated through a combination of medication and non-pharmacological treatments. With an efficacious treatment, it can reduce the risk of morbidity and mortality related to hypertension [3,4].

The improvement in hypertension of a patient requires him or her to consume the medication religiously. A common drug used is Metoprolol Tartrate, which is under the beta-blockers class of drugs [4]. A sudden stop in the consumption of this medication will in turn result in sharp chest pain, irregular heartbeat, or heart attack in serious cases. However, many patients might still find it challenging to comply with the course, as they are likely to be already taking multiple pills for other purposes. Moreover, this medication should be taken over a prolonged period of time, with possible side effects. The usage of this single drug does not provide a controlled release of the drug in the therapeutic range. Therefore, with a controlled release system, there will be better patient compliance and reduced side effects.

Globally, it is estimated that 1 billion people have hypertension, contributing to more than 7.1 million deaths per year [5,6]. Out of the 1 billion people, around 20% of the world’s adults have hypertension, where it prevails in patients older than 60 years and 70 million coming from American adults [7]. Furthermore, it has a high cost on the nation of $46 billion each year.

A common drug used has been Metoprolol Tartrate (MET) known as a beta-blocker. The improvement in hypertension of patients requires to take MET religiously. A sudden stop in the consumption of MET would result in sharp chest pain, irregular heartbeat, or heart attack in serious cases. In other words, the challenge of the use of MET is that the patient needs constant consumption of the drug. This is the reason that MET is used in immediate-release formulations [4], where it is absorbed quickly into the bloodstream. This often results in side effects and tissue toxicity, such as the liver. Furthermore, this problem of having a short half-life of most drugs and constant dosage requirement resulted in high plasma concentration peak and wearing-off in the concentration after the end of drug administration [4]. Thus, the problem of non-controlled release systems leading to under- and over-dosing of drugs, side effects, and decreasing patient compliance is an important topic to study on.

Nano- and microparticles have become an important tool for reformulating traditional pharmaceutical delivery systems. Especially, the biodegradable polymers such as poly (D,L-lactide-co- glycolide) (PLGA), poly(L-lactide) (PLLA), and polycaprolactone (PCL) have been used for the fabrications of particulate systems such as microcapsules or microspheres because of their biodegradability and biocompatibility, and the ability of controlled release [8,9,10,11,12]. The particulate systems allow for controlled release related to reduced dosing frequency and drug dosages. Furthermore, the controlled release profile of drugs could improve patient compliance and long-term therapeutic outcomes. However, PLGA-based particulate systems are not able to exhibit the high encapsulation efficiency of hydrophilic drugs due to their relative hydrophobicity [13]. 

**Scheme 1:**
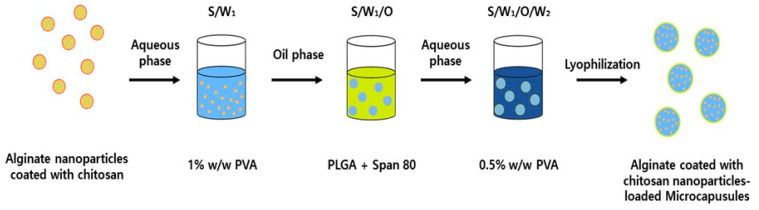
Schematic illustration of the encapsulation of metoprolol tartrate (MET)-loaded alginate coated with chitosan nanoparticles into poly (DL-lactide-co-glycolide) (PLGA) microcapsules.

Therefore, the aim of this work was to develop a novel drug delivery platform that enables enhanced encapsulation efficiency and controlled release with reduced initial burst release of the hydrophilic drug (MET) (Scheme 1). The release profiles of the drug can be changed by altering the degradation rate of the biopolymers used to fabricate the nanoparticle (NP). Here, we first report on the natural-derived hydrophilic polymer-based nanoparticles-encapsulating polymer microcapsules. As such, biocompatible and biodegradable polymers such as alginate and chitosan were chosen for the fabrication of nanoparticles, and PLGA was chosen for the fabrication of microcapsule (MC). The hydrophilic drug used for the medication of hypertension, MET, was chosen. The physicochemical properties such as drug encapsulation efficiency and the drug release rates were determined after the fabrication.

## 2. Materials and Methods

### 2.1. Materials

Alginic acid sodium salt from brown algae (low viscosity, Sigma-Aldrich, St. Louis, MO, USA) and Calcium chloride (Sigma-Aldrich, St. Louis, MO, USA) was used for cross-linking to achieve the NP. Chitosan (low molecular weight, Sigma-Aldrich) was used for the coating of alginate NPs. PLGA (50:50) and Polyvinyl alcohol (PVA) (molecular weight 30–70 kDa, Sigma-Aldrich, St. Louis, MO, USA) were used without further purification. MET (Sigma-Aldrich, St. Louis, MO, USA) was purchased directly from the supplier. Dyes used for confocal such as Trypan Blue (Dye content 60%) and Rhodamine B (≥95%, HPLC) were purchased from Sigma-Aldrich. Surfactant, Span 80 (nonionic surfactant), was purchased from Sigma-Aldrich. Solvents such as dichloromethane (DCM) were purchased from Tedia Co. Inc. (Fairfield, OH, USA). Buffer solution, Phosphate Buffer Saline (PBS) (pH 7.4, Sigma-Aldrich, St. Louis, MO, USA), was used as the release study medium.

### 2.2. Fabrication Methods

#### 2.2.1. Alginate NPs

Sodium alginate solution (10 mL) was pre-mixed with the hydrophilic drug (MET) (5%, 10%, or 20% *w*/*v* drugs). Calcium chloride solution (9 mM, 2 mL) was added drop-wise to the alginate solution while stirring at 800 rpm (Cross-linking). After an hour, the NPs were dialyzed overnight and freeze-dried.

#### 2.2.2. Nanoparticles-Encapsulated Microcapsules

The 10% (*w*/*w*) of MET-loaded alginate NPs were coated with chitosan. Briefly, chitosan (0.5 mg/mL) was dissolved in 1% of acetic acid and pH was adjusted to 4.0. The alginate NPs were incubated with chitosan solution for 15 min at 37 °C. The alginate NPs coated with chitosan were centrifuged and washed using DI water. NP suspension (100 mg/0.5 mL) was homogenized in 0.5 mL of PVA solution (1% *w*/*v*) homogenously by vortex mixer and ultra-sonication for 1 min. Span 80 (0.2 mL) was added to PLGA DCM solution (8% *w*/*v*) before adding NP suspension. For encapsulating the drug in microcapsules, drug was added into the polymer solution by homogenization using IKA Ultra-Turrax^®^ T25 at 10,000 rpm for 10 min. This polymer mixture was added to an aqueous solution (250 mL) containing PVA 0.5% (*w*/*v*) and emulsified at 500 rpm using an overhead stirrer at room temperature for 4 h. Then, particles were collected by centrifugation at 7000 rpm for 5 min and washed 2 times.

PLGA microparticles were fabricated by double emulsion solvent evaporation method. Briefly, MET (20 mg) was added to DI water (0.1 mL). PLGA (0.2 g) was dissolved in DCM (6.25 mL). Then, the two solutions were mixed under gentle stirring (250 rpm) to form a W1/O phase. The W1/O phase was transferred into 300 mL of PVA aqueous solution (5% *w*/*v*) and emulsified under overhead stirrer for 4 h at 500 rpm. The PLGA microparticles were collected by centrifugation (4000 rpm, 10 min), washed with DI water, freeze-dried, and kept at 20 °C for further studies.

### 2.3. Confocal Laser Scanning Microscopy (CLSM)

To have the strongest indications of the effectiveness of the encapsulation method, in-depth fluorescent imaging with CLSM can be used [14]. The alginate nanoparticles and chitosan were treated with fluorescent dyes to make the selected objects visible. Alginate and chitosan were treated with Rhodamine B (≥95%, HPLC) and Trypan Blue (Dye content 60%), respectively. The samples loaded with the fluorescent dyes were passed to the School of Biological Science for further analysis. CLSM was also able to obtain the distribution of the drugs loaded graphically.

### 2.4. Size and Zeta Potential Analysis

For the analysis of the particle size, Malvern Nanosizer (Zetasizer) was used. Approximately 1 mL of nanoparticle and water suspension was collected and poured into the cuvette for size analysis. The size and polydispersity index of alginate nanoparticle and alginate nanoparticle coated with chitosan were measured by dynamic light scattering using a 90° scattering angle and a temperature of 25 °C.

Zeta potential analysis of the chitosan coating on alginate nanoparticles was conducted using Malvern Nanosizer (Zetasizer). Approximately 1 mL of nanoparticle and water suspension was collected and inserted into a folded capillary cell using a syringe. The zeta potential of the standards, alginate, and alginate coated with chitosan was measured.

### 2.5. Encapsulation Efficiency and Drug Loading Efficiency

For the determination of encapsulation efficiency and drug loading efficiency of MET, approximately 10 mg of microcapsules were solubilized in DCM (1 mL). Next, distilled water (20 mL) was added to the microcapsule-solubilized solution. Then, the hydrophilic MET was extracted by ultrasonication for 10 min. The filtered supernatant was analyzed by high-performance liquid chromatography (HPLC) (Agilent 1200; Agilent Technologies, Santa Clara, CA, USA) with an autosampler and UV detector. UV-Vis detector was used with a wavelength of 233 nm. The column used was C18 (250 × 4.6 mm,5 µm) and the mobile phase contained 35% acetonitrile and 65% distilled water. Encapsulation efficiency and drug loading efficiency were calculated [15,16,17].

### 2.6. In Vitro Release Study

Three duplicates of 20 mg of each microcapsule sample (PLGA encapsulating alginate nanoparticles and PLGA encapsulating alginate nanoparticle coated with chitosan) were placed in 10 mL glass vials, which contain 10 mL of PBS (Phosphate Buffered Saline) pH 7.4 solution each. The six samples were then magnetically stirred over 22 days in a water bath at 37 °C. For every 1 mL of the medium being extracted from each bottle at a different time point, 1 mL of new medium was replaced. This ensures that there is a uniform volume of buffer solution in each glass vial over the drug release process. The buffer solution samples were extracted and the concentration of MET was determined using HPLC-UV method described above.

### 2.7. Change of Molecular Weight

Molecular weight of the microcapsules was measured using the Agilent GPC 1100 Series using a reflective index detector (RID) at 30 °C. Chloroform used as solvent and the flow rate was 1 mL/min. One mg of chloroform was added to dried PLLA and analyzed for GPC. Molecular weights of the microcapsules were calculated by the calibration curve using polystyrene standards (165–5000 kDa).

### 2.8. Statistical Analysis

Data were expressed as mean ± SD. Statistical analysis was conducted using GraphPad Prism 5.0 software (San Diego, CA, USA). The student’s t-test was used to compare two different groups of samples. Differences among multiple groups were performed by one-way ANOVA followed by Tukey’s multiple comparison. *p-value < 0.05, **p-value < 0.01 and ***p-value < 0.001 were considered significant.

## 3. Results

### 3.1. Physicochemical Properties

**Figure 1:**
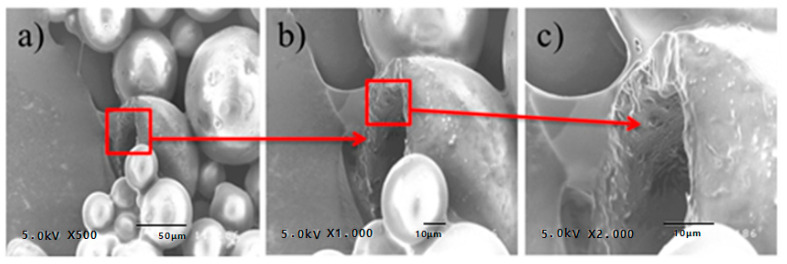
SEM images of (**a**) cross-section of microcapsule, (**b**) distribution of nanoparticle (NPs) encapsulated, and (**c**) zoomed-up image of NPs (Alginate NPs). Red square and arrow mean zoomed-up area of previous image.

**Figure 2:**
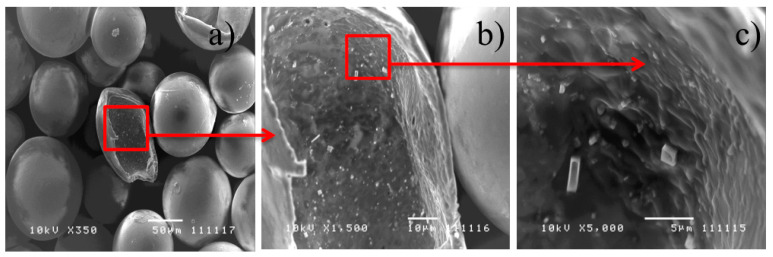
SEM images of (**a**) cross-section of microcapsule, (**b**) distribution of NPs encapsulated, and (**c**) zoomed-up image of NPs (Chitosan coated alginate NPs). Red square and arrow mean zoomed-up area of previous image.

The SEM cross-sectional images of alginate NP-loaded PLGA microparticle (MP) and alginate NP coated chitosan-loaded PLGA MP are shown in Figure 1 and Figure 2. To expose the interior structure, showing the nanoparticles encapsulated, there is a need to cut the particles using a scalpel. The typical size of the microcapsules achieved was approximately 100 µm. This can also help in the confirmation of the hollowness of the microcapsules. The images exhibited a hollow core filled with NPs. Regardless of the materials of NPs, all the NPs were localized near the capsule shell. This is further supported by the studies done, where the drug-loaded particles were successfully encapsulated in a hollow microcapsule [18].

SPAN 80 is a non-ionic ester of sorbitan oleate approved by FDA. It is used as a surfactant and has a Hydrophile-Lipophile Balance value of 4.3 [19,20]. This means that it is insoluble in water but soluble in the organic solvent [19,20]. In this study, SPAN 80 was used to separate the nanoparticles in the water phase from the microcapsule emulsion. As such, a higher amount of nanoparticles could be loaded into the microcapsule cavities.

An increase in the concentration of drugs from 1 mg to 4 mg results in a slight increase in the size of the nanoparticles from 114.2 ± 8.3 nm to 128.9 ± 4.3 nm (data not shown). This means that a higher amount of drugs, i.e., 4 mg, embedded into the alginate matrix, could induce a looser cross-linking structure of the drug-loaded alginate matrix. Furthermore, it can be observed that the concentration of alginate solution will also have an effect on the size of the particles [14,21].

The nanoparticle samples were subsequently encapsulated in the microcapsule. From the results reflected in Table 1, it can be observed that an increase in drug loading from 1 to 4 mg results in a minimal decrease in the encapsulation efficiency. This suggests that the quantity of sodium alginate becomes inadequate to entrap the drug [14,22].

### 3.2. Confocal Laser Scanning Microscopy (CLSM)

**Figure 3:**
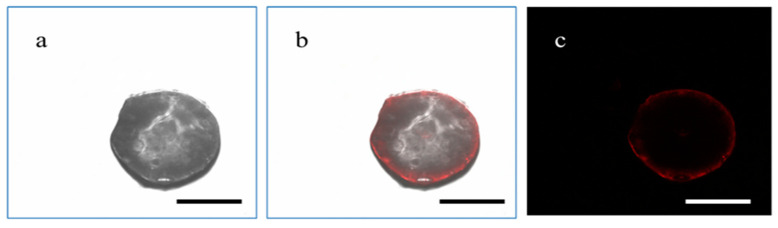
Confocal laser scanning microscopy (CLSM) images of alginate NPs-encapsulated microcapsule. (**a**) Brightfield, (**b**) Overlaid representation of all the components, and (**c**) Emission of the Rhodamine B(red) encapsulated in alginate NPs. Scale bar = 50 μm.

**Figure 4:**
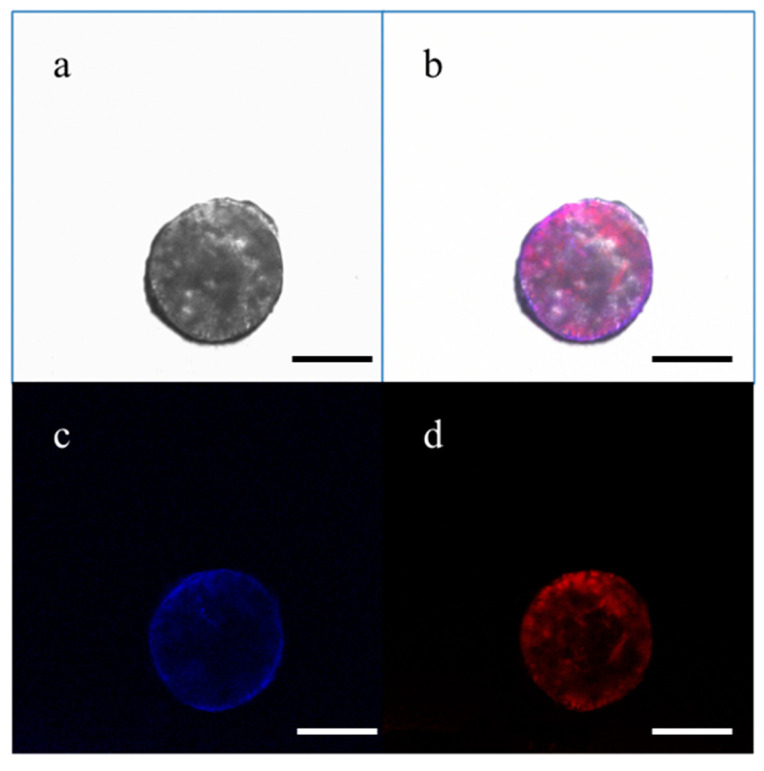
CLSM images of alginate coated with chitosan NPs-encapsulated microcapsule. (**a**) Brightfield, (**b**) Overlaid representation of all the components, (**c**) Emission of Trypan Blue(blue) loaded in chitosan layer and (**d**) Rhodamine B(red) loaded in alginate NPs. Scale bar = 50 μm.

**Figure 5:**
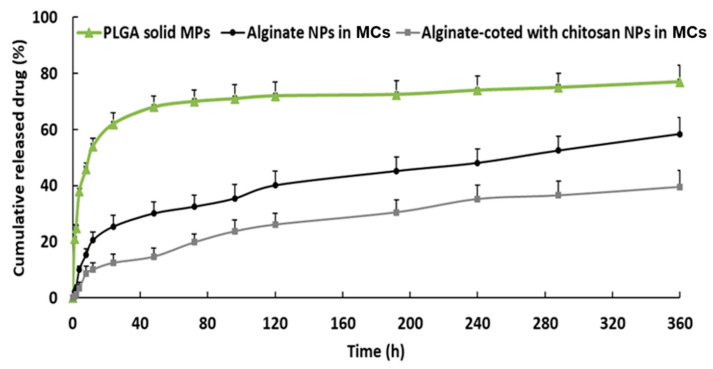
The cumulative release profile of MET from PLGA MPs, alginate NPs in MCs, and alginate coated with chitosan NPs in MCs (*n* = 3, mean ± SD).

Confocal Laser Scanning Microscopy (CLSM) was used to show the qualitative graphics on the distribution of the encapsulated particles. CLSM is very useful for determining the relative positions of particles or capsules in three dimensions. Therefore, we tried to show the successful encapsulation of nanoparticles in PLGA microcapsules by two different studies including SEM and CLSM. Different dyes (i.e., rhodamine B and trypan blue) were replaced by Figure 3 and Figure 4 show the CLSM images of the PLGA microcapsule with the encapsulation of dye-loaded alginate NPs and alginate NPs coated with chitosan, respectively. It can be seen from Figure 3b and Figure 4b that the fluorescence of the dyes did not transcend out of the PLGA MCs wall when the optical images (Figure 3c and Figure 4c,d) were overlaid with their respective brightfield images (Figure 3a and Figure 4a). The brightfield images in Figure 4 and Figure 5 exhibit irregular spheroid shapes. Since the microcapsule has a hollow core where nanoparticles are encapsulated, the microcapsule possesses an imperfect spheroid shape. That is the reason why the brightfield image looks overexposed. What the authors have tried to explain from CLSM images, the Rhodamine B encapsulated in nanoparticles were well-dispersed in the hollow core of the microcapsules. Furthermore, in Figure 3b and Figure 4b, the microcapsules had shown simultaneous emission spectra, proving that there was a successful encapsulation of the dye-loaded nanoparticles within a microcapsule. Thus, this method proved that the encapsulation of nanoparticles within microcapsule was successful. It can also show that the distribution of the nanoparticles was along the inner wall of the shell of the PLGA microcapsule. With all the results obtained from the CLSM, it could be concluded that NPs were successfully entrapped in the PLGA MCs.

### 3.3. In Vitro Release Study

As mentioned in the above section, the controlled and sustained release of hydrophilic drugs from PLGA microparticles is challenging due to the hydrophobic nature of PLGA polymer. To compare the release profiles of MET from conventional PLGA microparticles (MPs) and NPs-loaded PLGA MCs, in vitro release studies were conducted in PBS over 15 days (Figure 5). Figures S1–S3 exhibit three replicated of MET release profile from three different formulations.

**Figure 6:**
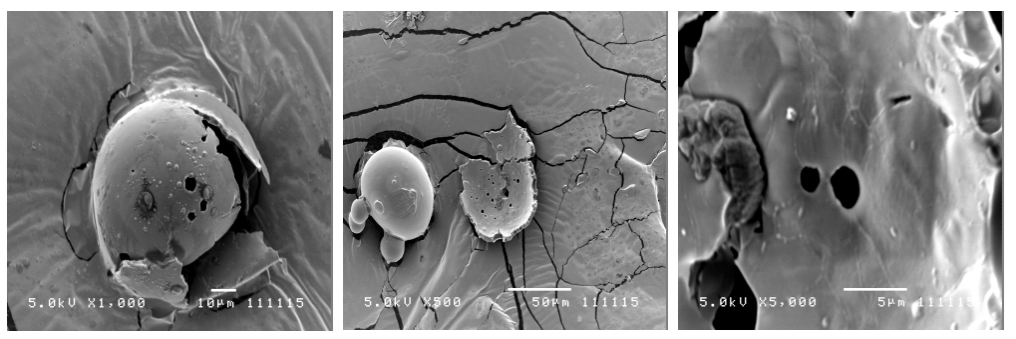
SEM images of degraded MC after 22 days of drug release.

**Figure 7:**
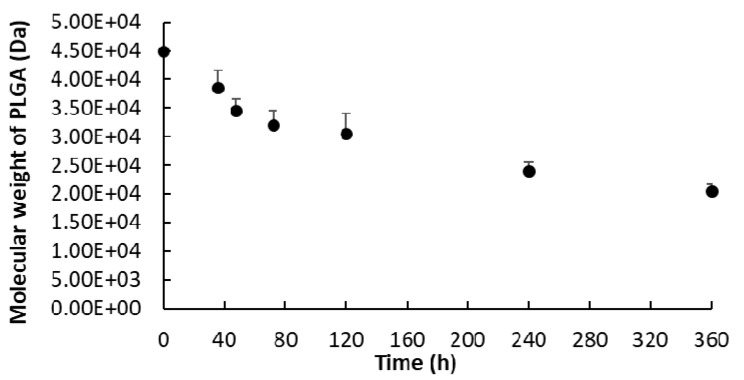
Change in molecular weight of PLGA of the degrading alginate-coated with chitosan nanoparticles loaded microcapsules as a function of incubation time (*n* = 3).

As expected, the cumulative amount of released MET from conventional PLGA MPs, which did not load NPs, was almost complete within five days. Despite the release of MET was not fully complete at 5 days, there was no further release of MET afterwards. On the other hand, the release profiles of MET from alginate nanoparticles in the PLGA MCs and alginate coated with chitosan NPs in MCs. The cumulative amounts of MET obtained were within the range of 55–90%, which is similar to other studies [23]. In another study [24], the encapsulation efficiency was around 58–80%, which has higher encapsulation efficiency due to a significantly larger mean size of the particles. From the release studies, PLGA MCs with the encapsulation of alginate NPs exhibited a more rapid release of drugs as compared to PLGA MCs encapsulating alginate coated with chitosan NPs. It was noted that the chitosan coating onto alginate NPs enabled to reduce the initial burst release of MET. When it comes to the cumulative amount of released MET at first 4 h, the released amount of MET of alginate NPs in MCs and alginate-coated with chitosan NPs in MCs was 10.2 and 3.5%, respectively, which was the almost three-fold difference. After the drug release study for 22 days, the particles were observed under SEM at 5.0 kV under different magnifications. From Figure 6, it can be observed that the appearance of particle surface was significantly changed and degradation was observed after 22 days in the PBS buffer medium. In addition, the molecular weight of PLGA decreased according to the release period, indicating the continuous degradation of PLGA microcapsules (Figure 7). Biodegradability is a very important property for biomaterials [25,26] to allow the release of drugs in a sustained manner.

In order to investigate the mechanism of the release profile of MET from PLGA MPs and two PLGA MCs, the release values were fitted to zero-order, first-order, and Higuchi’s equation (Figure S4) [27]. As for PLGA MCs, first-order equation exhibited the highest R2 values among the three equations, indicating the release mechanism depending on the drug concentration. On the other hand, the values of the PLGA MCs exhibited higher than that of PLGA MPs, indicating the mechanism of release profile was controlled-diffusion [18]. Both PLGA MCs would release the MET by the diffusion from alginate and chitosan matrix, while the drug was directly released from the outer rim of the PLGA MPs. In other words, the apparent release behaviors with the formulations can be justified by the chemical structures and the properties of alginate and chitosan [28,29]. Since chitosan has good film-forming properties, the coating of chitosan on alginate NPs reduces the swelling of the matrix and prolonged the drug release properties as compared to uncoated particles [29]. Subsequently, the hydrophilic drug can diffuse through the relatively hydrophilic PLGA MCs [30]. Nevertheless, both profiles showed a relatively controlled and sustained release with a reduced initial burst release at the early stage, within the first 12 h, and a decline in the release rate subsequently. Chitosan coating could further provide a sustained release profile of the hydrophilic drug. Therefore, the encapsulation of hydrophilic drug in alginate NPs and alginate NPs-coated with chitosan could allow the sustained and controlled release profiles with reduced initial burst release. The slightly different release profile of hydrophilic drugs from both MCs could be utilized for regulating the plasma concentration of hydrophilic drugs to achieve optimal therapeutic efficiency with minimum side effects.

## 4. Conclusions

In summary, the encapsulation of drug-loaded natural-derived nanoparticles into microcapsule is a novel study and has been proven to be feasible. The fabrication methods of both nanoparticles and microcapsules are modified to design a delivery system able to control the delivery of hydrophilic drug and maintain a sustained dosage of drugs in the human body. This can result in better patient compliance and reduces side effects of drugs as a therapeutic amount of hydrophilic drugs are released. Parameters, such as the polymer type, size of particles, and concentration of materials used can all affect the fabrication results and allow the calibration of an advanced drug delivery system to release the drugs.

Hence, the development of such modified fabrication of microcapsule encapsulating hydrophilic drug-loaded nanoparticles has produced a significant increase in a controlled and sustained release of the hydrophilic MET. To prove in vivo profile and efficacy of the microcapsule, in vivo animal studies would be further conducted.

## Figures and Tables

**Table 1:** Encapsulation efficiency (EE) and drug loading (DL) before and after loading NPs in microparticles (MPs) (*n* = 3, mean ± SD).

Feeding Amount of MET (mg)	PLGA MPs	Alginate NPs before Adding to PLGA MCs	Alginate NPs Coated with Chitosan before Adding to PLGA MCs	Alginate NPs Coated with Chitosan after Adding to PLGA MCs
1	35.1 ± 2.7%	79.1 ± 2.7%	75.2 ± 3.2%	-
	(DL: 0.35)	(DL: 0.79)	(DL: 0.75)	
2	33.6 ± 5.1%	74.4 ± 5.1%	73.1 ± 2.8%	-
	(DL: 0.67)	(DL: 1.48)	(DL: 1.46)	
4	28.2 ± 4.2%	71.3 ± 4.2%	70.3 ± 4.5%	65.2 ± 3.7%
	(DL: 1.1)	(DL: 2.85)	(DL: 2.81)	(DL: 1.83)
6	25.7 ± 3.4%	53.6 ± 3.4%	50.1 ± 6.1%	-
	(DL: 1.54)	(DL: 3.21)	(DL: 3.01)	
8	15.4 ± 4.5%	32.5 ± 4.5%	30.2 ± 4.7%	-
	(DL: 1.23)	(DL: 2.60)	(DL: 2.41)	

## Data Availability

Not applicable.

## Supplementary Materials

The following are available online at https://www.mdpi.com/1422-0067/22/6/2792/s1, Figure S1: The cumulative release profile of MET from PLGA MPs (three replicates), Figure S2: The cumulative release profile of MET from alginate NPs in MCs (three replicates), Figure S3: The cumulative release profile of MET from alginate-coated with chitosan NPs in MCs (three replicates), Figure S4: Kinetic analysis of drug release: (a) zero-order kinetics, (b) first-order kinetics, and (c) Higuchi kinetics.
