# Supplementary material for: Biodegradable Nanoparticles-Loaded PLGA Microcapsule for the Enhanced Encapsulation Efficiency and Controlled Release of Hydrophilic Drug
Source: Int J Mol Sci. 2021 Mar 10;22(6):2792. doi: 10.3390/ijms22062792 (PMC7998393; doi:10.3390/ijms22062792)
Supplement: Supplementary file 1 [file ijms-22-02792-s001.pdf]

[Supportin Information]

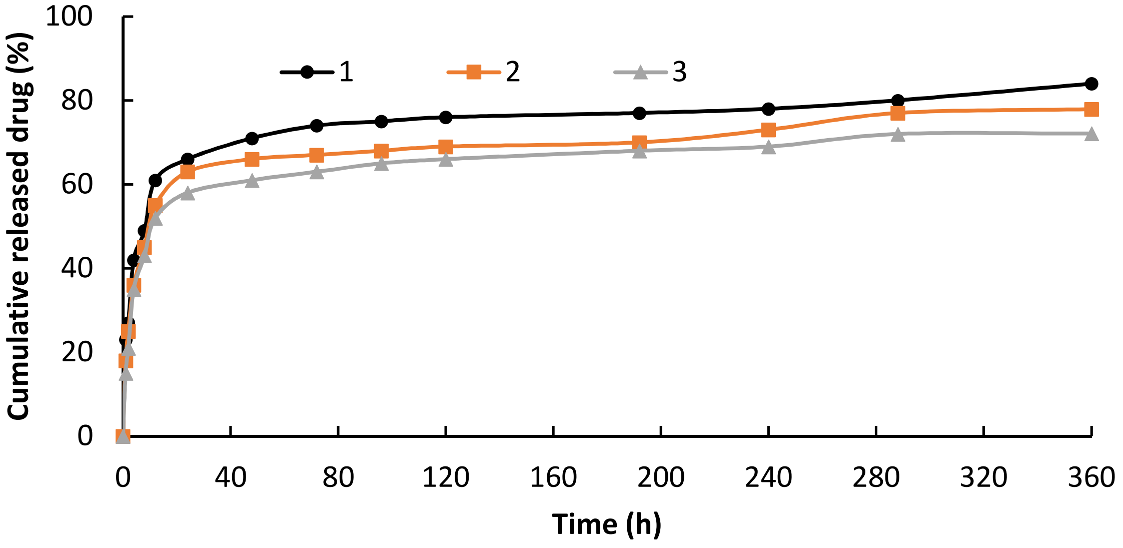

Figure S1. The cumulative release profile of MET from PLGA MPs (three replicates).

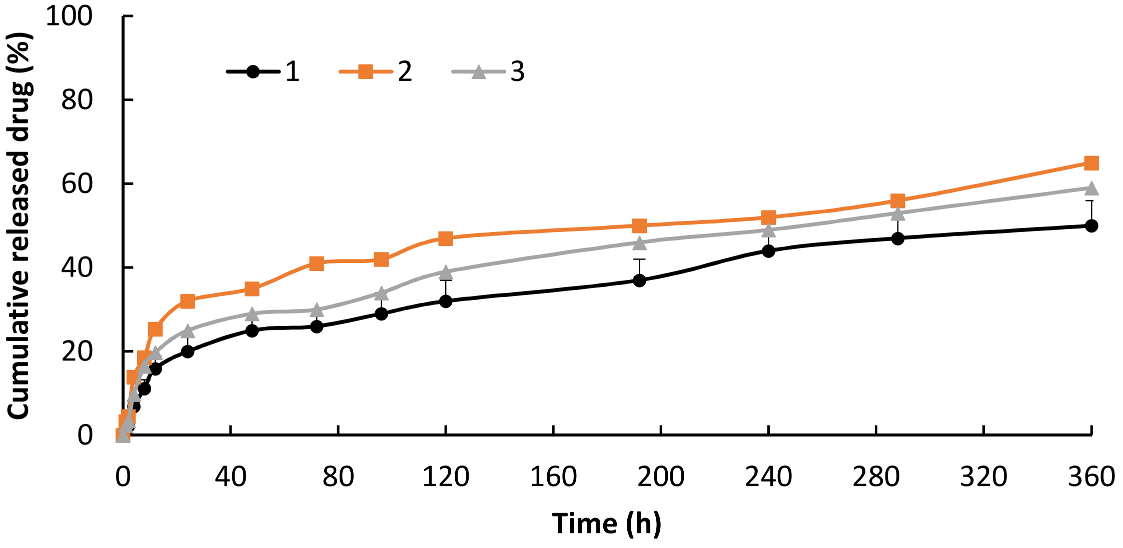

Figure S2. The cumulative release profile of MET from alginate NPs in MCs (three replicates).

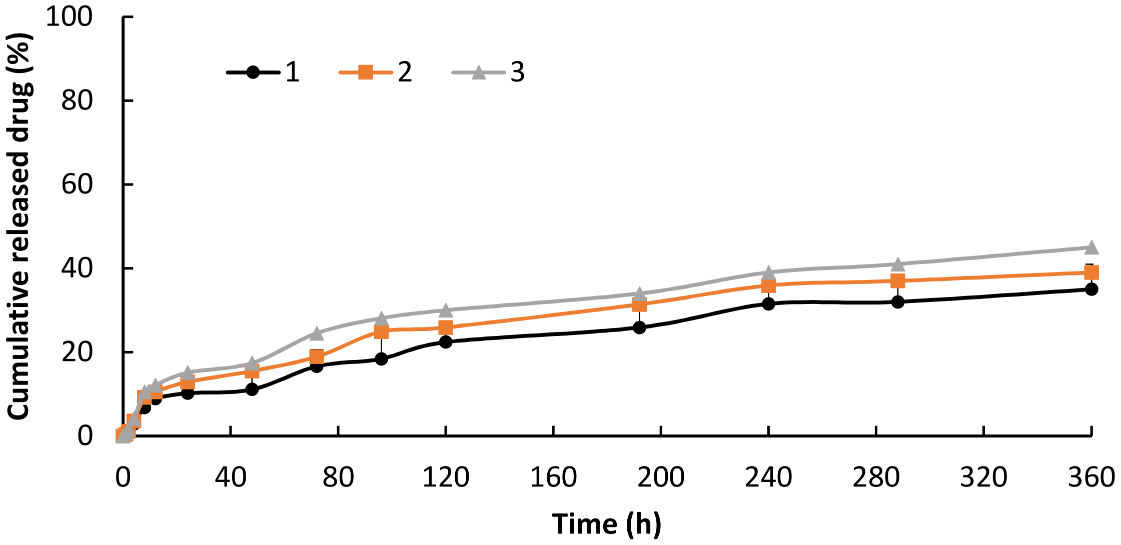

Figure S3. The cumulative release profile of MET from alginate-coated with chitosan NPs in MCs (three replicates).

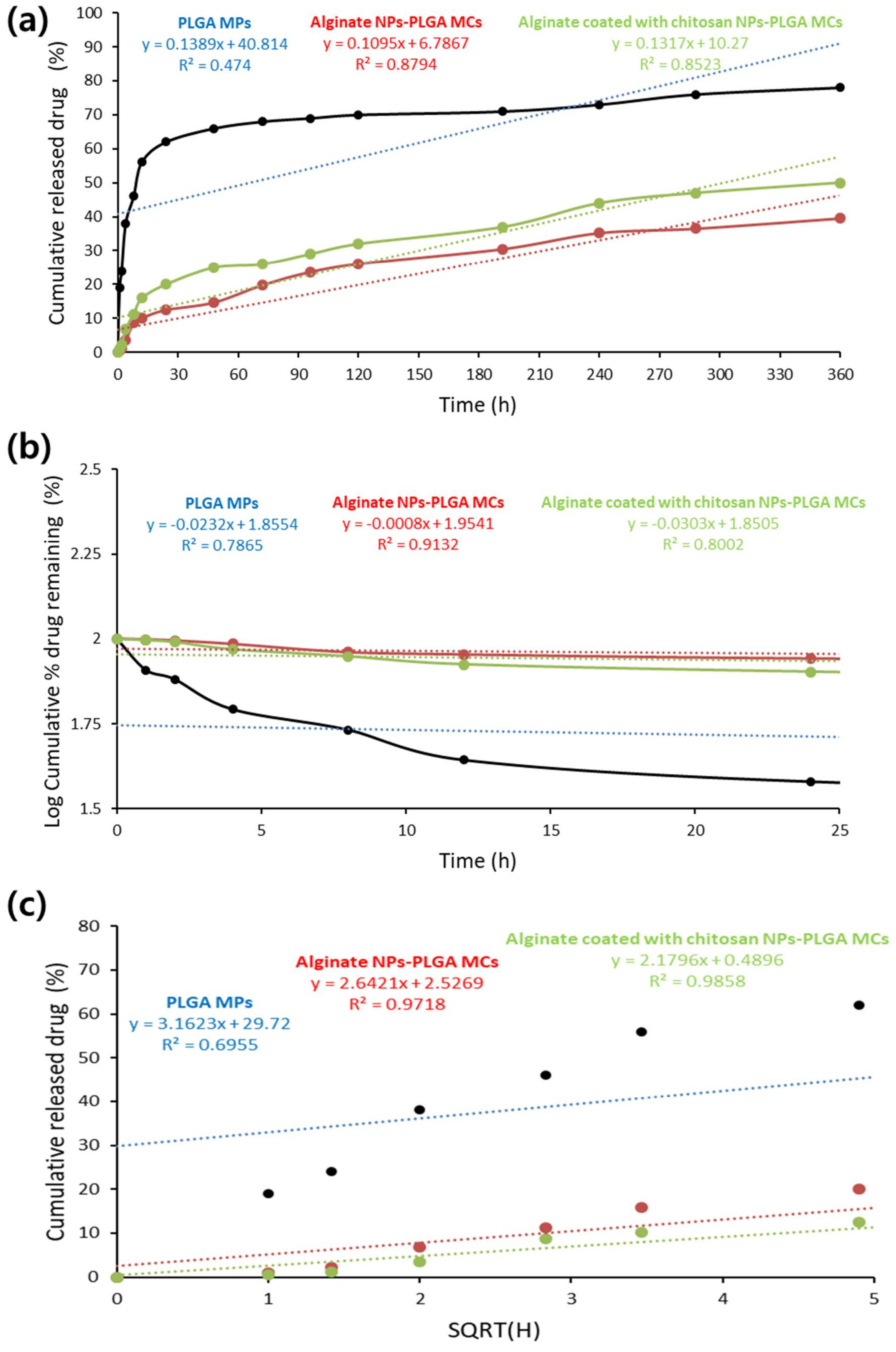

Figure S4. Kinetic analysis of drug release: (a)zero-order kinetics, (b) first-order kinetics, and (c)Higuchi kinetics.
